# Diversified Control Paths: A Significant Way Disease Genes Perturb the Human Regulatory Network

**DOI:** 10.1371/journal.pone.0135491

**Published:** 2015-08-18

**Authors:** Bingbo Wang, Lin Gao, Qingfang Zhang, Aimin Li, Yue Deng, Xingli Guo

**Affiliations:** 1 School of Computer Science and Technology, Xidian University, Xi'an, People’s Republic of China; 2 Institute of Software Engineering, Xidian University, Xi'an, People’s Republic of China; 3 School of Computer Science and Technology, Xi’an University of Technology, Xi'an, People’s Republic of China; University of Bonn, Bonn-Aachen International Center for IT, GERMANY

## Abstract

**Background:**

The complexity of biological systems motivates us to use the underlying networks to provide deep understanding of disease etiology and the human diseases are viewed as perturbations of dynamic properties of networks. Control theory that deals with dynamic systems has been successfully used to capture systems-level knowledge in large amount of quantitative biological interactions. But from the perspective of system control, the ways by which multiple genetic factors jointly perturb a disease phenotype still remain.

**Results:**

In this work, we combine tools from control theory and network science to address the diversified control paths in complex networks. Then the ways by which the disease genes perturb biological systems are identified and quantified by the control paths in a human regulatory network. Furthermore, as an application, prioritization of candidate genes is presented by use of control path analysis and gene ontology annotation for definition of similarities. We use leave-one-out cross-validation to evaluate the ability of finding the gene-disease relationship. Results have shown compatible performance with previous sophisticated works, especially in directed systems.

**Conclusions:**

Our results inspire a deeper understanding of molecular mechanisms that drive pathological processes. Diversified control paths offer a basis for integrated intervention techniques which will ultimately lead to the development of novel therapeutic strategies.

## Introduction

Network medicine [1] deals with complexity by simplifying cellular systems, summarizing them merely as biomolecular networks which are graphs with components (nodes) and interactions (edges) between them. There are different types of biomolecular networks such as genetic regulatory networks [2, 3], biochemical reaction networks [4, 5] and protein-protein interaction networks [6, 7] represent the functional, biochemical and physical interactions that can be identified with a plethora of technologies [8]. Network-based approaches to human disease take a complex disease stems as the malfunctions of corresponding biomolecular networks [9, 10]. Therefore, one of important tasks is to identify the effects of cellular interconnectedness on disease progression.

Recently, control theoretical tools of complex network have become a topic of active pursuit [11–20] and been successfully used to analyze biomolecular networks. Many dynamic properties of complex disease, mediated by the underlying cellular network, can be learned from the control effects exerted by genetic factors or drugs [20–24]. In particular, Liu et al. [11] introduce a maximum matching approach to predict minimum driver set (MDSet) nodes for the control of various biological networks. Additionally, Liu et al. [20] elucidate the principles behind biochemical network observability by offering the essential sensors in cell communication or biomarker design. Rajapakse et al. [21] examine various aspects of a genomic state-dependent dynamic network and elaborate on the controllability of genomic networks during processes of genomic reorganization. Wuchty [22] shows that MDSets of proteins are more likely to be essential, cancer-related and virus-targeted genes and closely related to bottleneck interactions, regulatory and phosphorylation functions, and genetic interactions. Melissa et al. [23] assess the output controllability of protein glycosylation in Chinese Hamster Ovary Cell for addressing the problem of glycosylation heterogeneity. Dealing with dynamic systems that respond to external inputs with specific output signals, these works have successfully achieved the important functional characteristics of MDSets nodes for the control of complex biological networks. Whereas they only focus on the control roles of nodes, an intriguing question, however, remains what exactly the control paths by which genetic factors perturb biological networks look like.

Therefore, we wondered whether control paths that are related with pathogenesis of the complex disease from the perspective of network medicine as well. Starting from an MDSet genetic factors whose time dependent control can guide the whole biological network to any desired final state, input control signals transmit along directed paths to all other genetic factors. These directed paths are called control paths. The dynamical process of propagating the perturbation influence relies on these control paths. We expected that the control paths carry biological significance, for example, disease-related pathway.

In this work, the control paths are defined on the maximum matching set (MMSet) edges which are a stem-cycle disjoint cover of the network and show us the directed paths along which the input control signals are transmitted. Moreover, diversified MMSets bring us diversified control paths (DCpaths) in which a node participates. For this node, the downstream reachable set nodes in these DCpaths are used to index its perturbation influence in this network. Focusing on the currently best investigated interactomes we determined the genes’ DCpaths in a human regulatory network. The known disease genes’ perturbation ranges were indeed enriched with disease-related pathways. Furthermore, DCpaths are used to analyse gene-phenotype relationship data from the Online Mendelian Inheritance in Man (OMIM) [25] and to test, by the leave-one-out cross-validation, the application in prioritizing candidates for all diseases with at least two known disease genes. The case studies of Alzheimer Disease, Diabetes Mellitus Type 2 and Leukemia strongly suggest that such well-defined DCpaths have significance in the identification of novel causal genes and disease pathways.

## Results

### Diversified control paths

According to Kalman’s controllability rank condition [26], a linear time-invariant dynamic system $\dot{X}\left(t\right)=A\cdot X\left(t\right)+B\cdot u\left(t\right)$ is controllable, if and only if the the *n* × *nm* controllability matrix *Q*_*C*_ has full rank, i.e.,

$$rank\left(Q_{C}\right)\equiv \left[B,AB,A^{2}B,\ldots ,A^{n-1}B\right]=n$$ (1)

where the state vector $X\in R^{n}$, $A\in R^{n}\times R^{n}$ is the adjacency matrix, $B\in R^{n}\times R^{m}$ is the input matrix, $u\in R^{m}$ is the input vector, *m* is the number of driver nodes and *n* is the number of nodes. The underlying directed network of this system is denoted by *G*(*A*), with node set *V* and link set *L*. But, it is computationally infeasible for complex networks to verify Kalman’s condition. To overcome this difficulty, Liu et al. [11] proposed the concept of maximum-matching set (MMSet) to assess and quantify structural controllability of arbitrary complex networks. A particularly useful result is the number of MDSet nodes (*N*_*D*_) required to fully control a network *G*(*A*) is max{*n* − |*M*|,1}. An MMSet is a link set *M* ⊆ *L* with maximum cardinality, and no two links in *M* may share a common starting node or a common ending node. |*M*| denotes the size of MMSet.

The controllability of a complex network concentrates on the interaction structure in which the pattern of influence may be known, but not the specific extent of influence [18]. In response to unknown or uncertain link weights, the controllability is used to uncover the generic properties of systems, independent of parameter values [27]. An MMSet shows the important links by which we can construct the cactus structures efficiently in a complex system [11]. The cactus must be the most economical topology-structure pattern to propagate control influence, since the cactus is a minimal structure such that removing any link will render the structure uncontrollable [28]. Therefore, we should recognize that the MMSet not only reveals the MDSet but also consists of a backbone of the key control paths. It forms a stem-cycle cover of the original network. Starting from the MDSet nodes input control signals transmit along the directed paths which are constructed by the MMSet links to guide the whole network to any desired final state. These directed paths are called control paths.

#### Definition 1

Control Path Set (CPSet) *C*_*k*_ is composed of the control paths which are connected by the links of a maximum-matching set *M*_*k*_ in a complex network.

**Fig 1:**
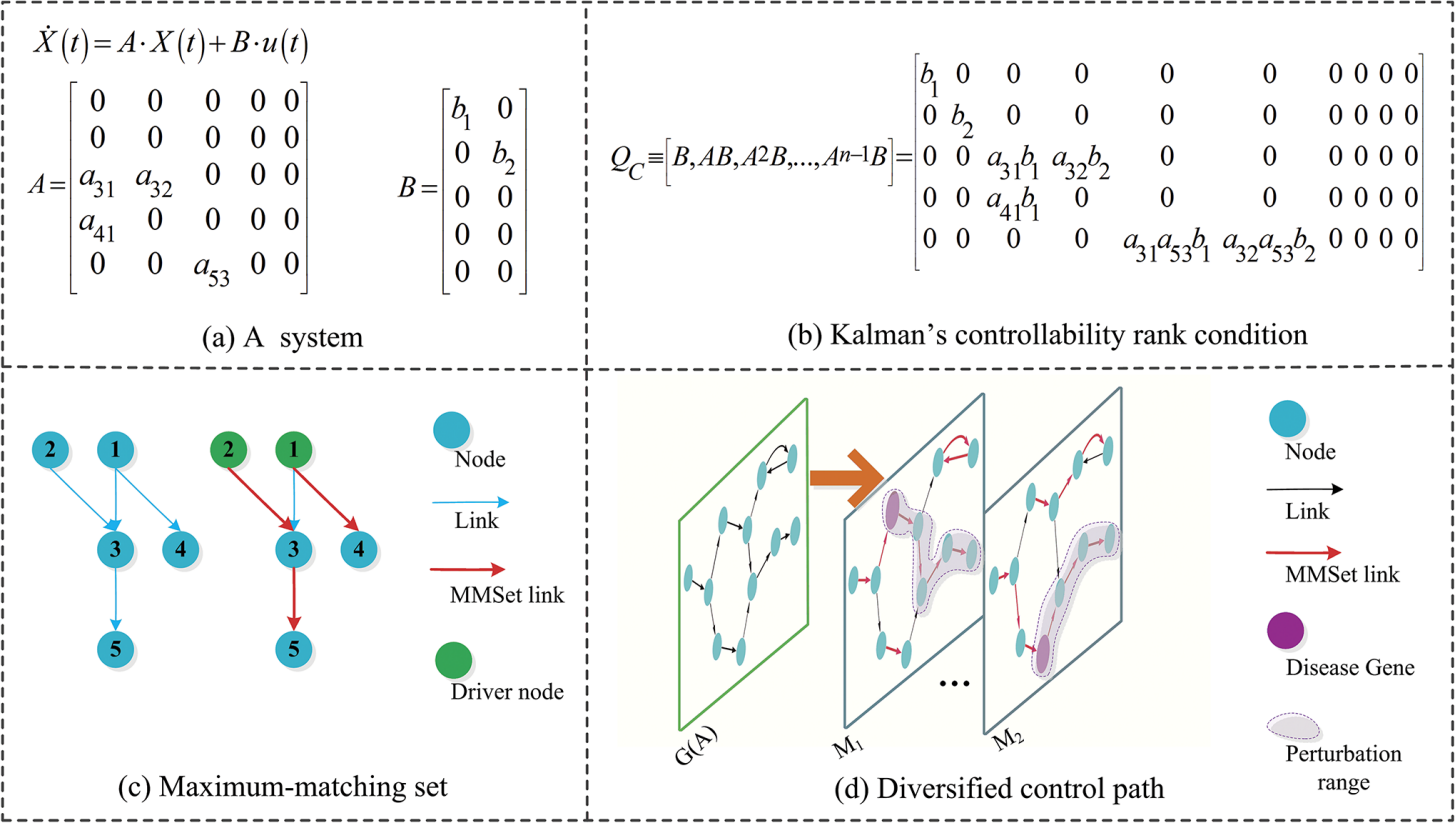
A schematic diagram of diversified control paths. (a): a linear dynamic system with adjacency matrix *A* and input matrix *B*. (b): the Kalman’s controllability rank condition, if *rank*(*Q*_*C*_) = *n*, this system is controllable. (c): for the underlying network, propose a maximum-matching set (MMSet) to assess the structural controllability. Links of the MMSet are highlighted by red. (d): for a network *G*(*A*), differentiated MMSet *M*_*1*_ and *M*_*2*_, marked by red, construct diversified control path sets. Disease genes are marked by purple and their perturbation ranges are indicated by shadow areas.

For example, a system with adjacency matrix *A* and input matrix *B* in Fig 1A, from the Kalman’s controllability matrix *Q*_*C*_ shown in Fig 1B, we can see the following important structural information for global controllability:

- If *a*_41_ = 0, *rank*(*Q*_*C*_) < *n*. Node 4 must only be influenced by node 1.
- If *a*_32_ = 0, *rank*(*Q*_*C*_) < *n*. When node 4 is controlled by node 1, node 3 must be influenced by node 2.
- If *a*_53_ = 0, *rank*(*Q*_*C*_) < *n*. Node 5 must be influenced by the state of node 3.
- If *a*_31_ = 0, *rank*(*Q*_*C*_) = *n* can be true. Without the influence coming from node 1, node 3 and node 5 can also be controlled.

Maximum matching is usually used to solve the assignment problem. Then we can also take the maximum matching as an assignment scheme of control influences in a complex network. Control influences are assigned according to an MMSet marked by red colour in Fig 1C, and the original network is divided into two control paths, through which all nodes can be controlled by the MDSet nodes marked by green colour. Losing some intricate control information between nodes inevitably, the MMSet absolutely retains all the four structural properties listed above and shows us the CPSet to govern the whole network. Therefore, we take the control paths as the significant pathways, implying critical topological information, which are related to the dynamical process of propagating the perturbation influence.

Furthermore, we note that, for a network there are diversified MMSets. Each one brings to our eyes a unique CPSet through which control influences transmit. The approach to enumerate diversified MMSets is given below. As showed in Fig 1D, for the network *G*(*A*), differentiated MMSet *M*_*1*_ and *M*_*2*_, marked by red colour, can shoulder the same control responsibilities and form diversified CPSets. The perturbation range (Pr) of a given node *i* under a MMSet *M*_*k*_ is a node set indicated as

$${\text{Pr}}_{i}\;\left(C_{k}\right)=\left\{j|\text{node}\;j\;\text{is reachable from node}\;i\;\text{through CPSet}\;C_{k}\;\right\}.$$ (2)

*k* = 1,2…*K*, *K* is the number of existing MMSets. Links in *M*_*k*_ invariably connect the nodes of Pr_*i*_ (*C*_*k*_) into a cactus structure originating from node *i*. Lin’s theorem [28] has demonstrated that a linear control system is structurally controllable if and only if the associated digraph can be spanned by cacti. So the states of nodes in Pr_*i*_ (*C*_*k*_) can be fully controlled by influencing node *i*. Two shadow areas in Fig 1D have displayed the perturbation ranges of two disease genes highlighted by purple.

#### Definition 2

Set $\underset{k}{⋃}C_{k}$ is the diversified control paths (DCpaths) of a complex network.

Then the perturbation influence (Pi) of a given node *i* can be indicated as

$${\text{Pi}}_{i}=\left\{j|\text{node}\;j\;\text{is reachable from node}\;i\;\text{through DCpaths}\;\right\}.$$ (3)

What we exactly want to do is use the perturbation influence, based on the control paths, to identify and quantify the ways by which disease genes perturb biological systems.

### Perturbation influence of disease genes

Firstly, we focus on how the known disease genes intervene in a biological system. The DCpaths of a human regulatory network (Table A in S1 File) is detected to reveal the perturbation influences of disease genes. Intuitively, for a disease, the overlap of disease genes’ perturbation influences can be taken as the significant pathways, which are related to its etiology essentially.

**Fig 2:**
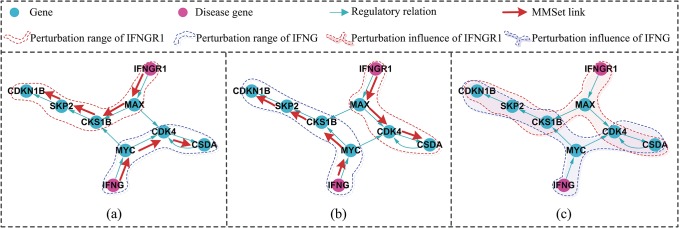
The perturbation influences of disease genes of the Tuberculosis (MIM:107470). (a): A CPSet of the partial human regulatory network, the MMSet links are highlighted by red, the disease gene IFNGR1 and IFNG are marked by purple, their perturbation ranges are circled with red and blue dotted lines respectively. (b): Another differentiated CPSet. (c): the perturbation influences of IFNGR1 and IFNG are marked by red and blue shadow respectively.

In Fig 2, the Tuberculosis (MIM:107470) in OMIM has 2 disease gene IFNGR1 and IFNG, which are also characterized by the partial regulatory network. Fig 2A and 2B show us two differentiated CPSets (highlighted with red) and the perturbation ranges of IFNGR1 and IFNG (circled with red and blue dotted lines respectively). Diversified control paths indicate the perturbation influences of IFNGR1 and IFNG (marked by red and blue shadow respectively) in Fig 2C. Their overlapped gene set {CDK4, CSDA, CKS1B, SKP2, CDKN1B} is considered as the potential pathways which have close relationships with pathogenesis of the Tuberculosis. All the five genes participate in the small cell lung cancer pathway (hsa05222 in KEGG [29]).

Also, we proceed to execute the DCpaths analysis on Thrombocythemia (MIM: 187950) and Immunodeficiency (MIM: 610163). The results are given in Table 1 with the disease name, disease gene list, genes’ perturbation influences and their common Gene Ontology (GO) terms [30]. For Thrombocythemia, the three known disease genes TPO, JAK2 and MPL can thoroughly perturb some common ranges which have the same biological functions, such as JAK-STAT cascade, growth hormone receptor signaling pathway, cytokine-mediated signaling pathway, etc. For Immunodeficiency, its known disease genes CD3E and CD3G almost have the same perturbation influences on the regulatory network, which chiefly affect the immune response of human.

**Table 1:** Instances for the perturbation influence of disease gene.

Disease	Disease gene	Perturbation influence	Common Gene Ontology term
Thrombocythemia(MIM: 187950)	TPO	JAK3, STAT1, SOCS1, IL20RB, TYK2, STAT4, SOCS4, CSF2RB, BAX	JAK-STAT cascade, Growth hormone receptor signaling pathway,Cytokine-mediated signaling pathway, …
	JAK2	STAT1, SOCS1, JAK3, IL20RB, STAT2, SOCS7, CRLF2	
	MPL	JAK3, STAT1, SOCS1, IL20RB, TYK2, STAT4, SOCS4, CSF2RB, BAX	
Immunodeficiency(MIM: 610163)	CD3E	ZAP70, CD3D, CD3Z, NCR3, FCER1G, NCR1, FCER1A, MS4A2, FCGR3	Innate immune response,Regulation of immune response,Regulation of immune effector process,Regulation of defense response,…
	CD3G	ZAP70, CD3Z, NCR3, FCER1G, NCR1, FCER1A, MS4A2, FCGR3	

It is clear that the DCpaths can be used to index the ways by which the disease genes influence pathological processes. And for the same disease, known disease genes’ perturbation influences are same and indeed enriched with disease-related pathways. To further demonstrate the power of DCpaths, we prioritize the candidate genes based on the assumption that the genes cause the same disease by driving the same perturbation influence in the human regulatory network.

### Prioritization of candidate genes

With investigation of the relative location of the candidate to all of the known disease genes by the use of perturbation influence, we assign a score to each of the candidate genes:

$$score(i)=\text{max}\;\left\{sim({\text{Pi}}_{i},{\text{Pi}}_{x})|x\in X_{d}\right\}$$ (4)

where, *X*_*d*_ is the set of known disease genes of disease *d*, for any given disease genes *x* ∈ *X*_*d*_, the biological functional similarity between the perturbation influence Pi_*i*_ and Pi_*x*_ is calculated. The maximum value of similarities is taken as the score of a gene *i* for disease *d*. The details of how the similarity is obtained are given below. Then the genes are ranked according to the score in order to define a priority list of candidates for further biological investigation.

In Table 2, Alzheimer Disease (MIM:104300), Breast cancer (MIM:114480) and Colorectal cancer (MIM: 114500) are proceeded for studies. Unsurprisingly, our method assigned the high ranks to the known disease genes in all cases. What is more, we detect some potential causal genes from the top ranked candidate genes as showed in Table 3. Most of them have been proved to be closely related to the corresponding dieases’ pathogenesis by the existing literatures. For instance, Kanekiyo et al. [31] have demonstrated that the low-density lipoprotein receptor-related protein 1 (LRP1) plays a critical role in brain amyloid-β (Aβ) peptides clearance and the Accumulation, aggregation, and deposition of Aβ are likely initiating events in the pathogenesis of Alzheimer’s disease (AD); Protein precursor cleaving enzyme 1 (BACE1) is the first protease and the rate limiting enzyme in the genesis of amyloid-β. This protein remains an important potential disease-modifying target for the development of drugs to treat AD [32]; Protein kinase C-alpha (PRCK1) regulates MDR1 expression with siRNA and reverse chemoresistance of ovarian cancer [33]; Epidermal growth factor (EGF) receptor is frequently overexpressed in the malignant phenotype of ovarian cancer leading to increased cell proliferation and survival [34]; AQP7 is a glycerol channel in adipose tissue with a suggested role in controlling the accumulation of triglycerides and secondly development of obesity and type-2 diabetes [35]; PIM-2 is a proto-oncogene and highly expressed in neoplastic tissues and in leukemic and lymphoma cell lines, the nuclear factor kappa B (NFKB1) pathway appears to be deregulated in a variety of tumors, with sustained activity of NFKB1 leading to apoptotic resistance in tumor cells [36].

**Table 2:** The ranks of known disease genes for three instances.

Alzheimer Disease (MIM:104300)					
Disease gene	Rank	Disease gene	Rank	Disease gene	Rank
APP	7	PLAU	4	A2M	2
NOS3	5	PSEN1	3	APOE	1
Breast cancer (MIM:114480)					
Disease gene	Rank	Disease gene	Rank	Disease gene	Rank
CDH1	6	TP53	4	ATM	2
PIK3CA	5	PPM1D	3	RAD53	1
Colorectal cancer (MIM: 114500)					
Disease gene	Rank	Disease gene	Rank	Disease gene	Rank
CTNNB1	19	PIK3CA	16	TP53	9
AXIN2	2	SRC	3	TGFBR2	4
APC	1	NRAS	7	BAX	18
CCND1	5	BRAF	6	PLA2G2A	10
EP300	13	DCC	14	FGFR3	17
BUB1	12	BUB1B	11	BCL10	8

**Table 3:** The top ranked candidate genes for some instances.

Disease	Candidate Gene	Rank
Alzheimer Disease (MIM:104300)	LRP1	6
Alzheimer Disease (MIM:104300)	BACE1	17
Ovarian cancer (MIM: 167000)	PRKC1	4
Ovarian cancer (MIM: 167000)	EGF	5
Diabetes Mellitus, Type 2 (MIM:125853)	AQP7	23
Leukemia (MIM:601626)	PIM2	13
Leukemia (MIM:601626)	NFKB1	26
Colorectal cancer (MIM:114500)	PECAM1	15
Amyloidosis (MIM:105200)	APBB1	1

**Fig 3:**
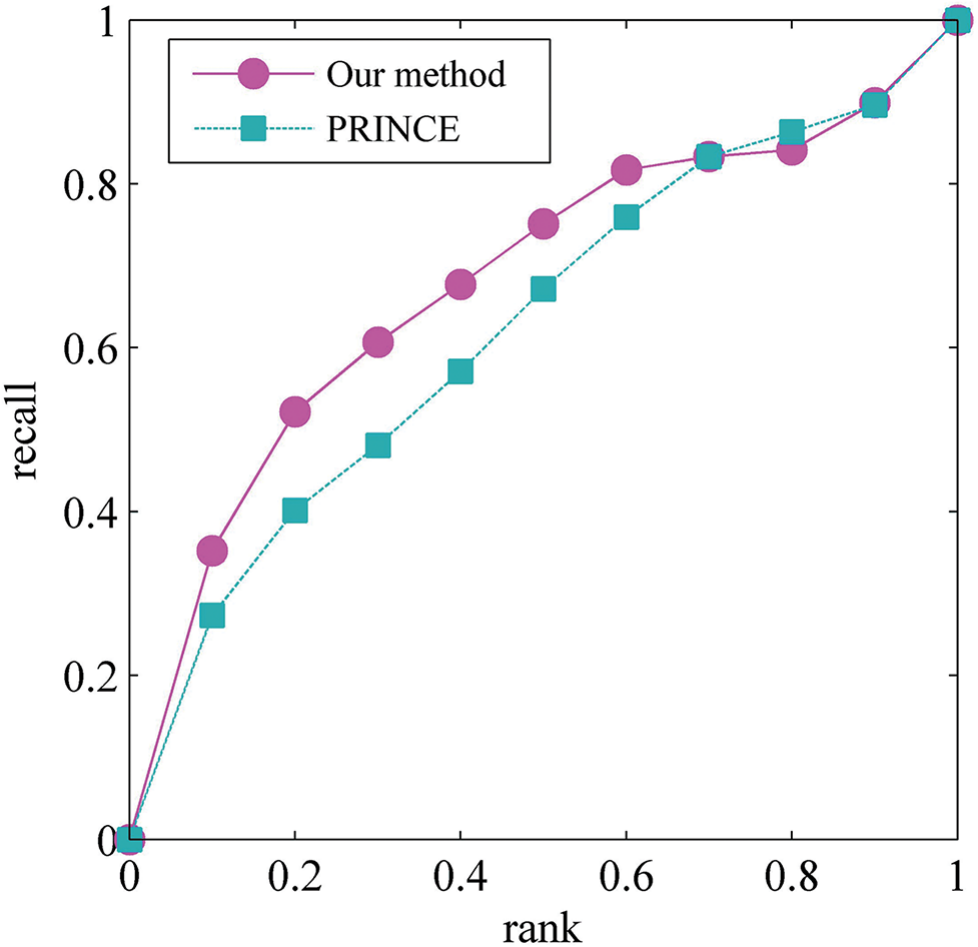
Comparison in performance between our method and PRINCE. A plot of recall versus rank threshold, rank threshold *k*% means that the gene was ranked within top *k*%.

We test our DCpaths-based method by Leave-One-Out Cross Validation (LOO-CV) [37]. Removing one disease-gene association in each cross validation trial, if this association can be ranked within top *k*% over the entire human regulatory network, it can be said that the association is reconstructed successfully. We evaluated prioritization results in terms of overall recall when varying the rank threshold *k*%. In Fig 3, the comparison with the sophisticated method PRINCE [38], obtained by prioritizing candidates on all 112 diseases in the LOO-CV, shows that our method achieve compatible prediction outcomes with PRINCE and further illustrate the disease genes perturb the biological system by the DCpaths we mentioned.

### Case study

Furthermore, to further demonstrate the significance of perturbation influences, we examine whether forecasted causal genes are enriched with disease pathways on multifactorial disorders or not. Alzheimer Disease (MIM:104300), Diabetes Mellitus, Type 2 (MIM:125853) and Leukemia (MIM:601626) are selected for case studies. We take the top 30 ranked candidate genes for these cases as the causal genes and check the metabolic pathways they participate in by GeneTrail [39]. Typical output of GeneTrail is a set of metabolic pathway terms with the size of the query and the term gene lists, their overlap gene lists and the statistical significance (p-value) of such enrichment.

Alzheimer Disease (MIM:104300) in OMIM gives a list of 6 known disease genes, which are also characterized by the human regulatory network. Besides these genes, the functional enrichment of other 24 causal genes within the top 30 are analysed in Table 4. We can see that 8 of them are involved in hsa04610: Complementand coagulation cascades (p-value = 4.69e-08), that 5 of them are involved in hsa05010: Alzheimer's disease (p-value = 6.04e-04), etc. Almost all the pathways are closely related with the current knowledge on Alzheimer Disease.

**Table 4:** Enrichment analysis of causal genes in Alzheimer Disease.

Metabolic pathway	P-value	Expected number of genes	Number of genes	Gene
hsa04610: Complementand coagulation cascades	4.69e-08	0.381904	8	PLAUR, PLG, F2, SERPINE1, F13B, FGA, F13A1, F11
hsa05010: Alzheimer's disease	6.04e-04	0.636507	5	LRP1, BACE2, GAPDH, BACE1, LPL
hsa04210: Apoptosis	2.60e-03	0.879537	6	AKT2, AKT1, AKT3, PRKACG, NFKB1, PRKX
hsa04914: Progesterone-mediated oocyte maturation	1.59e-02	0.856391	5	AKT2, AKT1, AKT3, PRKACG, PRKX
hsa05142: Chagas disease	2.14e-02	0.960547	5	AKT2, AKT1, AKT3, SERPINE1, NFKB1

Diabetes Mellitus, Type 2 (MIM:125853) in OMIM gives a list of 12 known disease genes, which are also characterized by the human regulatory network. Besides these genes, the functional enrichment of other 18 causal genes within the top 30 are analysed in Table 5. We can see that 8 of them are involved in hsa04930: Type II diabetes mellitus (p-value = 3.75e-09), that 7 of them are involved in hsa04960: Aldosterone-regulated sodium reabsorption (p-value = 5.43e-09), that 11 of them are involved in hsa04910: Insulin signaling pathway (p-value = 1.12e-08), etc. These agree well with the current knowledge on Diabetes Mellitus.

**Table 5:** Enrichment analysis of causal genes in Diabetes Mellitus, Type 2.

Metabolic pathway	P-value	Expected number of genes	Number of genes	Gene
hsa04930: Type II diabetes mellitus	3.75e-09	0.302998	8	PKLR, PIK3R3, PIK3R2, PIK3R1, PIK3R5, PIK3CG, PIK3CB, PIK3CD
hsa04960: Aldosterone-regulated sodium reabsorption	5.43e-09	0.208311	7	PIK3R3, PIK3R2, PIK3R1, PIK3R5, PIK3CG, PIK3CB, PIK3CD
hsa04910: Insulin signaling pathway	1.12e-08	1.13624	11	PKLR, PTPN1, EXOC7, TRIP10, PIK3R3, PIK3R2, PIK3R1, PIK3R5, PIK3CG, PIK3CB, PIK3CD
hsa04070: Phosphatidylinositol signaling system	3.11e-08	0.284061	7	PIK3R3, PIK3R2, PIK3R1, PIK3R5, PIK3CG, PIK3CB, PIK3CD
hsa04150: mTOR signaling pathway	1.79e-07	0.369279	7	PIK3R3, PIK3R2, PIK3R1, PIK3R5, PIK3CG, PIK3CB, PIK3CD

Leukemia (MIM:601626) in OMIM gives a list of 12 known disease genes, which are also characterized by the human regulatory network. Besides these genes, the functional enrichment of other 18 causal genes within the top 30 are analysed in Table 6. We can see that 8 of them are involved in hsa05221: Acute myeloid leukemia (p-value = 1.51e-07), that 7 of them are involved in hsa04662: B cell receptor signaling pathway (p-value = 3.41e-06), etc. These agree well with the current knowledge on Leukemia.

**Table 6:** Enrichment analysis of causal genes in Leukemia.

Metabolic pathway	P-value	Expected number of genes	Number of genes	Gene
hsa05221: Acute myeloid leukemia	1.51e-07	0.462914	8	PIM2, PIM1, RELA, NFKB1, PIK3R2, PIK3CG, PIK3CB, PIK3CD
hsa04662: B cell receptor signaling pathway	3.41e-06	0.530247	7	RELA, NFKB1, VAV2, PIK3R2, PIK3CG, PIK3CB, PIK3CD
hsa05220: Chronic myeloid leukemia	3.41e-06	0.521831	7	SHC2, RELA, NFKB1, PIK3R2, PIK3CG, PIK3CB, PIK3CD
hsa05212: Pancreatic cancer	3.41e-06	0.513414	7	ARHGEF6, RELA, NFKB1, PIK3R2, PIK3CG, PIK3CB, PIK3CD
hsa04062: Chemokine signaling pathway	2.83e-05	1.08574	8	SHC2, RELA, NFKB1, VAV2, PIK3R2, PIK3CG, PIK3CB, PIK3CD

### Robustness

To show the robustness of DCpaths, we test our method by prioritization of oncogenes, stability of perturbation influence of genes, and case study on Breast cancer (MIM: 114480) in a cancer signaling map (Table B in S1 File) [40]. The cancer signaling map was constructed by using cancer mutations and the literature-curated human signaling network. Characterizing an overall picture of the cancer, this network contains 326 nodes, 892 edges, in which 259 edges are indirected and we convert them into bi-directional edges. Then we extract the cancer signaling map with 326 nodes, 1151 edges to reveal the signaling architecture of cancer. 30 known cancer-gene associations are selected from the OMIM knowledge database.

**Fig 4:**
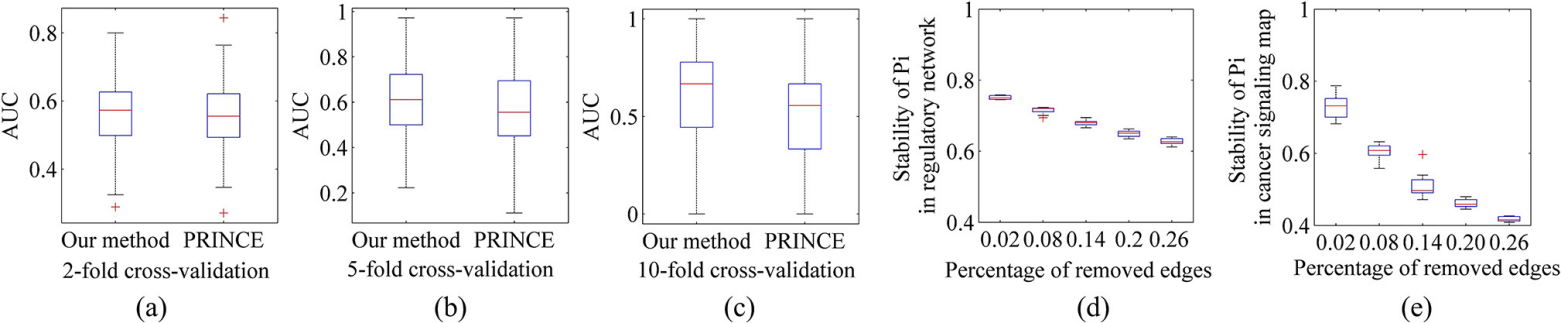
Analysis results for robustness of our method. (a) The AUC of 2-fold cross validation for prioritization of oncogenes in the cancer signaling map. (b) The AUC of 5-fold cross validation for prioritization of oncogenes in the cancer signaling map. (c) The AUC of 10-fold cross validation for prioritization of oncogenes in the cancer signaling map. (d) The stability of perturbation influence of the regulatory network. (e) The stability of perturbation influence of the cancer signaling map.

For prioritization of oncogenes, the 2-fold, 5 fold and 10-fold cross validation results have been provided in Fig 4A, 4B and 4C. Comparisons with PRINCE [38] on AUC [41] show that our method achieves better prediction outcomes than PRINCE. These results also embody the advantages of DCpaths in directed relationship analysis.

For stability of perturbation influence of genes, we remove a certain proportion of edges and assign a score to the stability of perturbation influence (SPi):

$$\text{SPi}=\frac{1}{n}\sum_{i}\frac{{\text{Pi}}_{i}\cap {\text{Pi}}_{i}\prime }{{\text{Pi}}_{i}\cup {\text{Pi}}_{i}\prime }$$ (5)

where, Pi_*i*_′ indicates the perturbation influence of a given node i after the a certain proportion of edges are removed. We take the Jaccard coefficient between the perturbation influences of node before and after deleting to measure the stability. Then the average of stabilities of all nodes is used to show the stability of perturbation influence of a network. In Fig 4D and 4E the SPi of the regulatory network and cancer signaling map are shown respectively, 20 times randomized experiments are conducted for each proportion. With the increase of the percentage of removed edges, the SPi goes down. But, on average, a node can maintain almost 70% original perturbation influence after 10% edges are removed in both the regulatory network and cancer signaling map. An MMSet experiences strong influence of removing, even the scale of MMSet will be changed. We use a random process to obtain variant MMSets as many as possible (see the next section). Based on diversified MMSets, DCpaths eliminates influences of changeful individual MMSet and Pi show preferable stability under removing.

**Fig 5:**
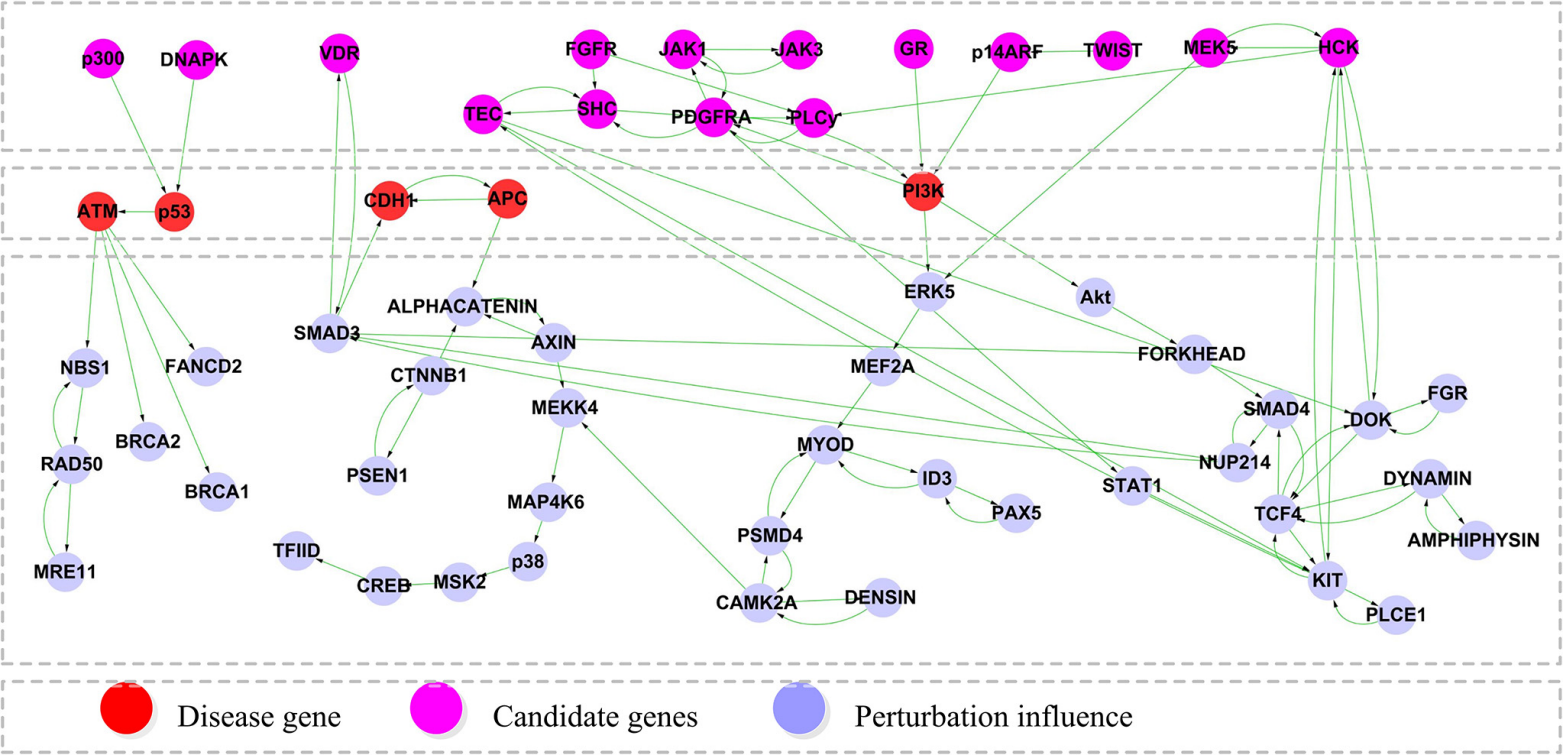
Sketch of prediction results for Breast cancer in Cancer Signaling Map. 5 known disease genes are highlighted by red color, 15 top ranked potential causal genes are highlighted by magenta color and the genes in the diversified control paths of disease genes are highlighted by purple color.

As a case study, we present the results of prioritization candidate genes on Breast cancer (MIM:114480) in the cancer signaling map. Breast cancer in OMIM gives a list of 5 known disease genes (APC, ATM, p53, PI3K and CDH1), which are further characterized by the cancer signaling map. Besides these genes, some potential causal genes from the top ranked candidate genes as showed in Table 7. Furthermore, we have downloaded the somatic mutations for Breast cancer (Table C in S1 File) from TCGA [42]. Most all of potential causal genes in Table 7 mutate in more than 2 samples. Having obvious mutation, they tend to play an important role in Breast cancer. Fig 5 vividly display the perturbation influence of disease genes by showing a subnetwork of the cancer signaling map. This subnetwork contains 5 known disease genes (highlighted by red color), 15 top ranked potential causal genes (highlighted by magenta color) and the genes (highlighted by purple color) in the diversified control paths of disease genes. These directed paths identify the ways by which 5 disease genes perturb the cancer signaling map. And the 15 potential causal genes also have ability to perturb these pathways, for they possess their own directed control paths lead to the perturbation influence of disease genes.

**Table 7:** The top ranked candidate genes for Breast cancer in Cancer Signaling Map.

Breast cancer (MIM:114480)					
Gene	Rank	Number of mutation samples	Gene	Rank	Number of mutation samples
APC	1	8	FGFR	11	4
ATM	2	20	GR	12	3
p53	3	265	PDGFRA	13	4
PI3K	4	2	JAK3	14	6
CDH1	5	58	JAK1	15	4
DNAPK	6	13	HCK	16	1
p300	7	8	VDR	17	1
p14ARF	8	2	TEC	18	3
TWIST	9	2	MEK5	19	2
SHC	10	1	PLCy	20	0

## Materials and Methods

The DCpaths-based approach requires a directed network as input. In this study, we consider a human KEGG regulatory network (Table A in S1 File), constructed by Backes et al. [3]. This network contains the regulatory relationships selected from all KEGG pathways and can be download from the website http://genetrail.bioinf.uni-sb.de/ilp/Home.html. Backes et al. access the data via the Biochemical Network Database (BNDB) [43] for a consistent interface. It contains 2010 genes connected by 9900 regulatory relationships, among which 1579 genes, annotated by GO terms, with 7630 regulatory relationships are selected to form our human regulatory network. The GO annotation is essential to the calculation of biological functional similarity between the perturbation influences. Our Leave-One-Out Cross Validation process needs one disease has at least 2 known disease genes. Therefore, 366 known disease-gene associations, satisfied this condition, are chosen from the OMIM knowledge database, relating 252 known disease genes to 112 diseases.

### Diversified MMSets enumeration

For a given directed network, anyone of the existing algorithms [44, 45] can be used to compute an MMSet. The Markov process, as described by Jia et al [46], performs unbiased random sampling among all MMSets and can be used to estimate the role of each vertex in controlling the network. We used the approach of Wang et al [47] to enumerate diversified MMSets. Beginning from an MMSet, randomly chooses a link in this MMSet, enumerates all alternative MMSets that include all other elements except this link, then randomly chooses one of these MMSets as the current MMSet and repeats the process. The DCpaths of our results is achieved for 827 diversified MMSets in 18649 random samples for the prioritization of candidate genes in the human regulatory network.

### Functional similarity between the perturbation influences

Then we present the detailed description of the algorithm to calculate the biological functional similarity between the perturbation influence Pi_*i*_ and Pi_*x*_ of given gene *i* and *x*.

**Algorithm** for *sim*(Pi_*i*_, Pi_*x*_):

**Input** the gene set Pi_*i*_ and Pi_*x*_

Construct a bipartite graph *BP*(Pi_*i*_, Pi_*x*_, *E*), ∀*u* ∈ Pi_*i*_, ∀*l* ∈ Pi_*x*_, *E*(*u*, *l*) = *GOSim*_*BMA*_ (*u*, *l*) [48]

Solve the *Maximum Weight Bipartite Matching Problem* on *BP* by the Hungarian algorithm [41].

**Output** the sum of the weights of the maximum matching as *sim*(Pi_*i*_, Pi_*x*_).

The perturbation influences of gene *i* and *x* are the gene set Pi_*i*_ and Pi_*x*_, we detect the best matching between these two gene sets, and use the GO annotation similarity to quantify the functional similarity of each matching pair (*u*, *l*). The similarity of two genes, indicated as *GOSim*_*BMA*_ (*u*, *l*), is computed by the method of Wang et al. [48]. The more genes in Pi_*i*_ having consistent functions with the genes in Pi_*x*_, the higher value *sim*(Pi_*i*_, Pi_*x*_) achieves.

## Conclusions

Medium-scale subnetworks, such as motif and community, represent the functional structures of a complex system. MMSet decomposes a network into medium-scale structures (stems and cycles), which are the subsistent control paths, by which we can control the whole system with the minimum driver nodes effectively. Therefore, detecting the significant DCpaths to quantify the perturbation influences of the genetic factors in the biological system is our goal. Although the influences of genetic factors are complicated and confused, DCpaths is an effective mean to analyze the intricate control relationship between them. To verify the power of DCpaths, we have handled the prioritization of candidate genes in the human regulatory network to analyze the perturbations of known disease genes, predict causal genes and detect disease pathways.

Using DCpaths to analyze pathogenesis is due to its several considerable merits: DCpaths give us a chance to understand the complex disease form a new perspective that how and to which extent does a genetic factor influences the network; DCpaths’ calculation has nothing to do with the weights of the regulatory relationships; DCpaths-based method can reveal very important functional relationships between genetic factors, which can not be detect by common neighbour or reachable path based methods, especially in directed biological systems. For instance in Fig 2, the disease genes IFNGR1 and IFNG of Tuberculosis have no common neighbours or reachable paths to each other, but their same influence on the dynamic properties of this disease can be uncovered based on DCpaths in the human regulatory network.

## Supporting Information

S1 File The experimental data. The human KEGG regulatory network (**Table A**), the cancer signaling map (**Table B**) and the somatic mutations for Breast cancer from TCGA (**Table C**).
(XLS)
